# A Randomized and Controlled Research Study Assessing the Emotions and Beliefs of Future Middle School Science Teachers toward Terrestrial Isopods

**DOI:** 10.3390/insects13030233

**Published:** 2022-02-26

**Authors:** Ron Wagler, Amy Wagler

**Affiliations:** 1 STEM Education Division, Department of Teacher Education, The University of Texas at El Paso, 500 West University Avenue, Education Building 601, El Paso, TX 79968, USA; 2 Department of Mathematical Sciences, The University of Texas at El Paso, 500 West University Avenue, Bell Hall 311, El Paso, TX 79968, USA; awagler2@utep.edu

**Keywords:** arthropod education, belief, crustacean education, disgust, fear, middle school science, terrestrial isopod

## Abstract

**Simple Summary:**

Terrestrial isopods are small land-dwelling animals and can be an effective curriculum tool when used in a science classroom. A study was performed where future middle school science teachers participated in activities with living terrestrial isopods. We found that terrestrial isopods are the ideal “model” living arthropod to initially use in middle school science teacher preparation programs and middle school science teacher professional development.

**Abstract:**

Terrestrial isopods, a diverse group of small crustaceans, are a beneficial component of a healthy ecosystem. Terrestrial isopods are also excellent living animals to have in a middle school science classroom. The current study evaluated if future middle school science teachers would utilize living terrestrial isopods in their classroom, and if they would not, to what extent fear and disgust towards arthropods was a factor that influenced their decision to avoid them. Before the terrestrial isopod activities, the teachers had moderate fear and moderate disgust toward terrestrial isopods and had no desire to teacher their students about terrestrial isopods. After participating in the terrestrial isopod activities, the teachers had no fear and no disgust toward terrestrial isopods and had a strong desire to teach their students about terrestrial isopods. Based on the findings of this study, new discoveries and powerful recommendations are presented that are relevant to those that are involved in the preparation of future middle school science teachers and those that provide professional development for current middle school science teachers.

## 1. Introduction

Arthropods make up over 75% of all animal species on Earth [1]. Some examples of arthropods that people frequently encounter include cockroaches, spiders, flies, and beetles. Based upon sheer numbers, global biodiversity, and evolutionary history, arthropods are arguably the most biologically successful animal phylum that has ever existed and are involved in a myriad of ecological processes that make human existence possible [2]. Even though this is the case, it has been well documented that people tend to find many species of arthropods disgusting and fear them [3,4,5,6,7,8]. When these emotions are elevated in modern life, this can lead to the individual avoiding certain events or activities [9,10].

**Figure 1:**
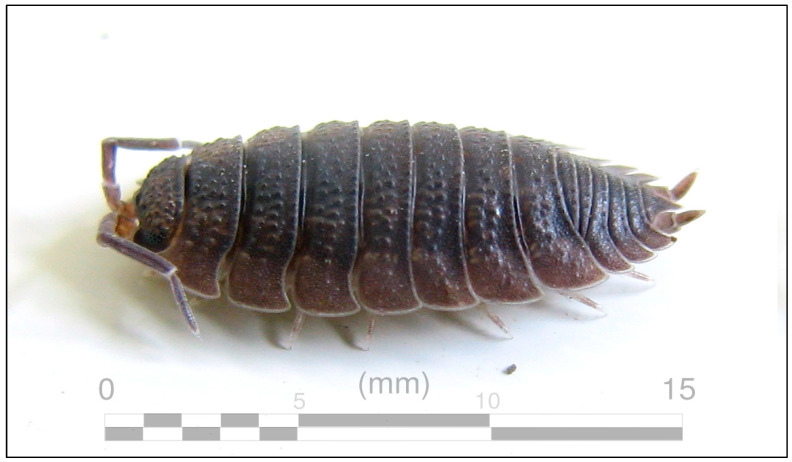
An Adult *Porcellio scaber*. Creative Commons: https://en.wikipedia.org/wiki/Porcellio_scaber#/media/File:Porcellio_scaber_(AU)-left_01.jpg, (accessed on November 29, 2021).

One of the major groups of arthropods are crustaceans (shrimp and lobsters), with most species being aquatic. Terrestrial isopods (See Figure 1), a diverse group of small crustaceans with approximately 3700 species, are an exception since they live on land [11]. These animals can regularly be found under leaf litter and rocks throughout much of the planet and are often referred to as roly polies, sow bugs, pill bugs, and many other common names. Even though terrestrial isopods are small, their numbers are many, and they participate in numerous natural processes that strengthen the health of Earth’s ecosystems. Ecological services that terrestrial isopods participate in and perform include: eating dead plants and dead animals; increasing soil nutrients by participating as detritivores; and being food for other animals in food chains. Because of these characteristics, terrestrial isopods are a beneficial component of a healthy ecosystem.

Terrestrial isopods are also excellent living animals to have in a middle school science classroom, and prior research has shown that when children work with these animals, their level of fear and disgust toward terrestrial isopods declines [12]. Some of their positive attributes include simplicity of care, a quick reproductive rate, low cost, a gentle nature, and their use as an effective tool for teaching students many different general science concepts, biology concepts, and ecology concepts. The current study evaluated whether future middle school science teachers would utilize living terrestrial isopods in their classroom, and if they would not, to what extent fear and disgust towards arthropods was a factor that influenced their decision to avoid them. The study employed a randomized and controlled research design and occurred in a college level science methods course with future middle school science teachers. The study investigated how activities with living terrestrial isopods affected the emotions of fear and disgust teachers held toward terrestrial isopods, their beliefs concerning the likelihood of incorporating information about terrestrial isopods into their science classroom (LITI), and their beliefs concerning the likelihood of incorporating information about the ecological services of terrestrial isopods into their science classroom (LIETI). See Wagler and Wagler 2016 [13] for a complete description of the studies’ theoretical underpinnings (human belief, human disgust, and human fear) presented in a science classroom context.

Beyond the positive attributes terrestrial isopods provide to science classrooms, they are also ideal arthropods to use for this kind of study because they participate in ecological services, and an effective middle school science curriculum using terrestrial isopods is currently available [14,15]. Lastly, this is an important cohort of teachers to evaluate because after elementary school students learn about basic ecology content such as food chains, they progress on to middle school where they need to learn more complex ecology content related to ecosystem processes, ecosystems, and biodiversity. If middle school science teachers are not properly trained on how to teach this more complex science content to their students, these students will enter high school with a limited understanding of ecology.

## 2. Materials and Methods

### 2.1. Research Questions

1. Did the terrestrial isopod activities change the teachers’ levels of fear toward terrestrial isopods?
2. Did the terrestrial isopod activities change the teachers’ levels of disgust toward terrestrial isopods?
3. Did the terrestrial isopod activities change the teachers’ beliefs concerning LITI?
4. Did the terrestrial isopod activities change the teachers’ beliefs concerning LIETI?

### 2.2. Procedure

The subjects of the study were future middle school science teachers that were part of a science methods course at a large United States (U.S.) university. None of the teachers had taken an invertebrate biology or entomology course and all were non-science majors. The study utilized a randomized pretest/post-test design with a control group. The teachers were randomly placed into either the control group or the treatment group. There were 103 teachers in the treatment group and 101 teachers in the control group. The treatment group had 97 females and 6 males with an average age of 27.3. The control group had 93 females and 8 males with an average age of 28.1. Both the control group and the treatment group were given a pretest at the start of the semester. The teachers were shown a living adult terrestrial isopod (*Porcellio scaber*) (see Figure 1) in a round clear plastic container (11.43 cm dia. × 4.13 cm H) without a lid. Each teacher was permitted to view the terrestrial isopod from an estimated distance of 20 cm for no longer than 60 s.

The teachers then rated their level of fear toward the terrestrial isopod (Likert scale: No Fear (1) to Extreme Fear (5)) and level of disgust toward the terrestrial isopod (Likert scale: No Disgust (1) to Extreme Disgust (5)). The teachers then rated their LITI (Likert scale: Extremely Unlikely (1) to Extremely Likely (4)) and their LIETI (Likert scale: Extremely Unlikely (1) to Extremely Likely (4)). After this, the treatment group participated in the terrestrial isopod activities, but the control group did not. The terrestrial isopod activities were conducted for the duration of the semester. At the semester’s end, both the treatment group and the control group completed the post-test, which was identical to the pretest.

### 2.3. Overview of the Terrestrial Isopod Activities

**Figure 2:**
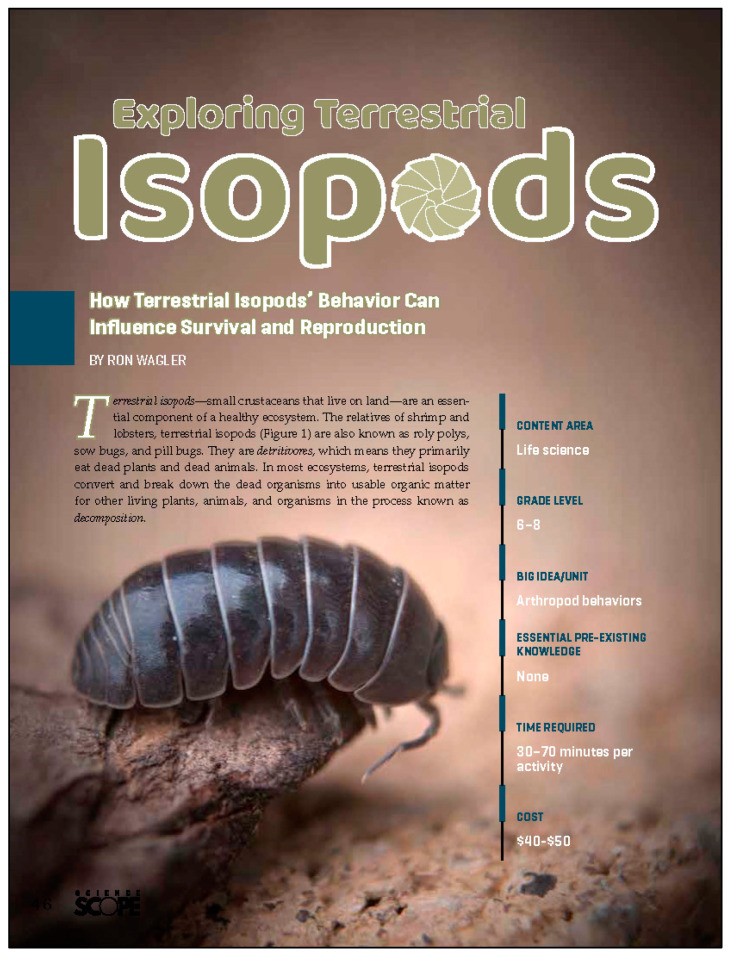
Exploring Terrestrial Isopods: Example Hands-on Inquiry Based Terrestrial Isopod Activities used in the Study.

Only the teachers in the treatment group participated in the terrestrial isopod activities. The terrestrial isopod activities were from the articles “The Wonders of Terrestrial Isopods” [14] and “Exploring Terrestrial Isopods: How Terrestrial Isopods’ Behavior can Influence Survival and Reproduction” [15] (See Figure 2). All the terrestrial isopod activities that were used in the study were from the journal *Science Scope,* and some of the activities were aligned to the *Next Generation Science Standards* [16,17]. The terrestrial isopod activities presented information about terrestrial isopod biology and information about the ecological services terrestrial isopods perform in Earth’s ecosystems. The terrestrial isopod activities were initiated at the start of the semester directly after the pretest was given. They were then performed during the semester and were completed immediately before the post-test was given at the semester’s end. The terrestrial isopod species *Porcellio scaber* (see Figure 1) was chosen for the study because they are frequently bred in captivity; are readily available; can safely be used in a science classroom; can be bought in the U.S.; and can be easily combined into the terrestrial isopod activities that were used in this current study [14,15]. Additionally, this terrestrial isopod species and these terrestrial isopod activities were selected so that after the studies’ participants were taught how to use the curriculum, they could easily implement the curriculum in their classroom.

### 2.4. Statistical Analysis

The study outcomes of fear, disgust, LITI, and LIETI were assessed for changes using cumulative logistic regression models [18]. The cumulative logit models will assess the association of each outcome with the treatment group and test period in order to investigate treatment- and time-based changes in the ratings. In the results, the model-based log odds ratios compare the likelihood of a positive increase in the rating for a teacher in the treatment group compared to a teacher in the control group at a specific time point. The models include two-way interaction between treatment group and time and a random effect for person. All the model results are reported with *p*-values that are adjusted for multiplicity using the Šidák correction [19]. The mean group by time comparison were assessed using model-based least squares means and using a Šidák multiplicity correction [19]. The studies’ analysis was completed by utilizing the ordinal package [20] in R [21].

## 3. Results

Table 1 presents summary statistics about the fear, disgust, LITI, and LIETI study endpoints. Using model results reported in Table 1, there was no evidence of a difference in the fear ratings for the control or treatment groups at the pretest point. After the intervention, there was a decrease in fear ratings for the treatment group, but a change was not observed in the control group (Z = 8.31, *p*-value < 0.0001). Similar results were observed for the disgust ratings. After the intervention, there was a decrease in disgust ratings for the treatment group, but again, a change was not observed in the control group (Z = 8.10, *p*-value < 0.0001). These results provide robust evidence that the treatment group participants experienced a decrease in disgust, whereas those in the control group show no evidence of a decrease.

With respect to the LITI outcomes, the treatment group participants had increased levels, whereas the control group did not change significantly (Z = 8.29, *p*-value < 0.0001). This indicates that the treatment group had much higher LITI ratings for terrestrial isopods, whereas the control group did not have a significant increase. The LIETI ratings increased for the treatment group participants (Z = 8.48, *p*-value < 0.0001), showing that these teachers had higher LIETI ratings for terrestrial isopods than the control group.

### Findings

The first two research questions assessed whether the terrestrial isopod activities changed the teachers’ levels of fear and disgust toward terrestrial isopods. Before the activities, both groups (treatment and control) had moderate fear and moderate disgust toward terrestrial isopods (See Table 1). After participating in the terrestrial isopod activities, the treatment group had no fear and no disgust toward terrestrial isopods, whereas the control group continued to have moderate levels of fear and disgust toward terrestrial isopods (See Table 1).

The last two research questions assessed whether the terrestrial isopod activities changed the teachers’ beliefs concerning LITI and LIETI. Before the terrestrial isopod activities, neither group (treatment and control) planned to incorporate information about terrestrial isopods or information about the ecological services of terrestrial isopods into their science classroom. After the terrestrial isopod activities, the treatment group had high levels of desire (Extremely Likely) to teach their students about the animals and the ecological services they perform (See Table 1). The ratings of the control group did not change and the teachers in this group had no interest in teaching their students this new science content. Note that further research is needed to evaluate how long the positive attributes of decreased fear and disgust and increased LITI and LIETI persist in middle school science teachers.

## 4. Discussion

Research conducted in the field of arthropod education that involves middle school science teachers is extremely limited. Only two previous studies have been conducted. Both of these prior studies, one with living cockroaches and one with living spiders, showed that middle school science teachers avoided teaching students about cockroaches and spiders and the important role they play in nature because they found these animals disgusting and feared them. The overall trend observed was that as fear and disgust towards arthropods increased, so too did the tendency to avoid teaching information about arthropods. Both studies also showed that through positive educational interventions with those living animals, these barriers could be overcome [22,23].

This study provides further validation supporting the findings of these two previous studies but also presents new discoveries and powerful recommendations for those that are involved in the preparation of future middle school science teachers and those that provide professional development for current middle school science teachers. Although the two previous studies showed that educational interventions with living cockroaches and living spiders could influence teachers to use living arthropods in their classrooms, neither were as highly effective as this study that used terrestrial isopods. Unlike the two previous studies where the teachers entered the study with higher levels of fear and disgust, the subjects of this study entered the study with lower levels of fear and disgust. In a practical sense, this means that the teachers were not initially as fearful and disgusted by the terrestrial isopods as they were by cockroaches and spiders in the two previous studies. Since these emotions were initially lower, the educational intervention was much more effective. The end result was that after the teachers completed the activities, their levels of fear and disgust towards the terrestrial isopods were much lower than what has been observed with prior interventions with living cockroaches and living spiders. Furthermore, the teachers’ desire to bring these amazing animals into their classroom and teach their students about them and why they are beneficial to nature was also much higher than has been seen with interventions with living cockroaches and living spiders. These are very promising findings.

Lastly, prior research studies have shown that humans have different attitudes and emotions depending on the type of arthropod [24,25] with spiders being one of the most feared arthropods [26]. The current study (along with Wagler and Wagler, 2018 [22] and Wagler and Wagler, 2021 [23]) validates the findings of these three prior research studies by showing that middle school science teachers also have differing attitudes and emotions toward specific types of arthropods (terrestrial isopods versus spiders versus cockroaches), but of these three arthropod types, they too have the greatest level of fear towards spiders. These fears, which also exist toward cockroaches but are not as high as with spiders, directly influence middle school science teacher’s desire to avoid spiders and cockroaches [27].

### Recommendations

Based on this study’s findings, it is recommended that terrestrial isopods be the first living arthropod used in middle school science teacher preparation programs and middle school science teacher professional development. By doing this, there will be a much greater chance that the teachers will incorporate terrestrial isopods into their middle school classroom versus if the instructor were to initially use living arthropods that illicit greater levels of fear and disgust such as cockroaches and spiders. Once terrestrial isopods have been used in the course and the students’ levels of fear and disgust have dropped to the level of minimal to no fear, then other arthropods that tend to illicit greater levels of fear and disgust such as cockroaches and spiders can then be introduced if time allows. In a scenario such as this, the students will be much more willing to work with living cockroaches or living spiders if their levels of arthropod fear and disgust have already been greatly reduced by the initial activities with terrestrial isopods.

Furthermore, it is recommended that programs that prepare middle school science teachers and those that provide middle school science teacher professional development use living terrestrial isopods first simply because of the amount of time, money, and space needed to incorporate these animals into a program versus using living cockroaches or living spiders. As previously mentioned, terrestrial isopods require much less care than cockroaches and spiders and also require much less space to house. They also require less equipment, cost less to feed, and have a much quicker reproductive rate than cockroaches or spiders. This quicker reproductive rate will allow the instructor to use the terrestrial isopods much sooner in their courses and also provide them with the large number of terrestrial isopods they will need for courses that have high student enrollment. Lastly, an effective peer reviewed terrestrial isopod care article and two middle school science curriculum articles using terrestrial isopods are currently available and can be requested from the first author for free. All these reasons make terrestrial isopods the ideal “model” living arthropod to initially incorporate and begin using in middle school science teacher preparation programs and middle school science teacher professional development.

## 5. Conclusions

Arthropods are essential to the health of global ecosystems, and without them, humans would quickly become extinct [2]. Recent research has shown that the largest group of arthropods, insects, are in serious global decline [28]. Knowing that the number of humans on the planet will soon pass 8 billion, and that humanity finds itself in the midst of a self-induced mass extinction, which has the potential to cause the collapse of the very ecosystems that sustain us, there is not a more pressing time to act. Terrestrial isopods, though small and seemingly insignificant, are the ideal “model” living arthropod for initial introduction into a classroom setting and can play a meaningful role in educating a new generation of students that can begin to place special emphasis on protecting the disappearing ecosystems that arthropods live and work in.

## Figures and Tables

**Table 1:** Mean (Standard Deviations) for Fear, Disgust, LITI, and LIETI Ratings.

Group	Time	Fear	Disgust	LITI	LIETI
Control	Pretest	3.01 (0.74)	3.05 (0.82)	2.21 (0.68)	2.22 (0.73)
Treatment	Pretest	3.03 (0.91)	3.02 (0.95)	2.20 (0.69)	2.21 (0.70)
Control	Post-test	3.02 (0.72)	3.06 (0.81)	2.19 (0.61)	2.21 (0.70)
Treatment	Post-test	1.52 (0.59)	1.50 (0.58)	3.71 (0.50)	3.72 (0.49)

## Data Availability

The data is not housed on a public website to protect the confidentiality of research participants. De-identified data is available upon request.
